# Early oxidative shifts in mouse skeletal muscle morphology with high‐fat diet consumption do not lead to functional improvements

**DOI:** 10.14814/phy2.12149

**Published:** 2014-09-17

**Authors:** Melissa M. Thomas, Karin E. Trajcevski, Samantha K. Coleman, Maggie Jiang, Joseph Di Michele, Hayley M. O'Neill, James S. Lally, Gregory R. Steinberg, Thomas J. Hawke

**Affiliations:** 1 Department of Pathology and Molecular Medicine, McMaster University, Hamilton, Ontario, Canada; 2 Department of Medicine, McMaster University, Hamilton, Ontario, Canada

**Keywords:** Diet‐induced obesity, exercise testing, fiber typing, metabolism, morphology

## Abstract

Short‐term consumption of a high‐fat diet (HFD) can result in an oxidative shift in adult skeletal muscle. However, the impact of HFD on young, growing muscle is largely unknown. Thus, 4‐week‐old mice were randomly divided into sedentary HFD (60% kcal from fat), sedentary standard chow (control), or exercise‐trained standard chow. Tibialis anterior (TA) and soleus muscles were examined for morphological and functional changes after 3 weeks. HFD consumption increased body and epididymal fat mass and induced whole body glucose intolerance versus control mice. Compared to controls, both HFD and exercise‐trained TA muscles displayed a greater proportion of oxidative fibers and a trend for an increased succinate dehydrogenase (SDH) content. The soleus also displayed an oxidative shift with increased SDH content in HFD mice. Despite the aforementioned changes, palmitate oxidation rates were not different between groups. To determine if the adaptive changes with HFD manifest as a functional improvement, all groups performed pre‐ and postexperiment aerobic exercise tests. As expected, exercise‐trained mice improved significantly compared to controls, however, no improvement was observed in HFD mice. Interestingly, capillary density was lower in HFD muscles; a finding which may contribute to the lack of functional differences seen with HFD despite the oxidative shift in skeletal muscle morphology. Taken together, our data demonstrate that young, growing muscle exhibits early oxidative shifts in response to a HFD, but these changes do not translate to functional benefits in palmitate oxidation, muscle fatigue resistance, or whole body exercise capacity.

## Abstract (precis)

Young, growing animals consuming a short‐term high‐fat diet (HFD) exhibit morphological and metabolic changes akin to that of exercise‐trained mice. We hypothesized that these changes may manifest in a functional outcome that was similar to the exercise‐trained mice. Despite these muscle adaptations with HFD consumption, no improvement in exercise or in situ muscle performance was observed, suggesting other limiting factors (e.g., decreased capillary density) in the HFD mice.

## Introduction

The consumption of a high‐fat diet (HFD) induces a shift toward a more oxidative profile (morphologically and enzymatically) in adult skeletal muscle (Hancock et al. 2008; de Wilde et al. 2008; Shortreed et al. 2009; Trajcevski et al. 2013). For example, 3 weeks of HFD consumption in adult mice resulted in increased IIa and decreased IIb fiber types, no change in mitochondrial enzymes, and an increase in palmitate oxidation (Trajcevski et al. 2013). However, despite this early oxidative shift in skeletal muscle with HFD, continuation of the diet for a further 5 weeks led to insulin resistance, a build up of intramuscular lipids (IMCL), and significant reductions in glucose and palmitate oxidation rates (Shortreed et al. 2009; Trajcevski et al. 2013).

The prevalence of obesity and its associated comorbidities (e.g., type 2 diabetes mellitus or T2DM) has seen dramatic increases over the last 30 years, with the rates among American and Canadian adolescents tripling over this time frame (Public Health Agency of Canada 2011; Karnik and Kanekar 2012). Historically, the presence of T2DM in adolescents has been virtually nonexistent, however, a recent study demonstrated that T2DM accounts for ~22% of all cases of youth diabetes (American Diabetes Association 2014). Despite this alarming trend, the young (pediatric) population is often overlooked in animal research studies with most research focusing on adult rodents after skeletal muscle growth has ceased. Of note, mice experience a doubling of muscle fiber cross‐sectional area between the ages of 3 and 8 weeks (White et al. 2010). Furthermore, between 3 and ~12 weeks of age, fast, glycolytic muscles decrease their relative expression of slow myosin heavy chain while slow oxidative muscles increase their relative expression of slow myosin heavy chain (Agbulut et al. 2003). Given these significant changes in skeletal muscle morphology and metabolism during development, it is possible that changes in diet composition could have more profound effects on young, growing muscle.

The research on human adolescents shows that obese adolescents have greater intramyocellular lipid (IMCL) content than nonobese adolescents (Sinha et al. 2002; Saukkonen et al. 2010) and that there is a correlation between higher IMCL levels and lower insulin sensitivity (Sinha et al. 2002). Skeletal muscle mitochondrial function has been reported to be decreased in obese insulin‐resistant adolescents while obese noninsulin‐resistant adolescents had no impairments in mitochondrial function (Slattery et al. 2014). Skeletal muscles from adolescent humans undergo changes with obesity that could affect muscle oxidative function.

Thus, the purpose of this study was to determine if young, growing mice fed a HFD (with 60% kcal from fat) for 3 weeks would exhibit oxidative shifts in skeletal muscle, akin to that observed in adult mice. Here, we present evidence that short‐term HFD consumption in young, growing mice leads to significant increases in body and epididymal fat masses, but no change in skeletal muscle masses or whole‐body insulin resistance. Within skeletal muscle, HFD consumption did result in adaptive responses, such as transitioning fiber types from fast, glycolytic (IIb) toward fast, oxidative (IIa) and increasing oxidative metabolism enzyme content (e.g., succinate dehydrogenase [SDH]). Although these changes to skeletal muscle composition were adaptive to handling a greater lipid load they did not result in a greater capacity for ex vivo palmitate oxidation rates.

A secondary purpose of this study was to investigate whether these early oxidative shifts would manifest as functional improvements on a progressive treadmill exercise test or in situ muscle function performance. Interestingly, despite a morphological shift in fiber‐type composition and an increased SDH content, there was no improvement (nor decrement) in maximal exercise capacity using a rodent treadmill or in response to fatiguing stimulations using in situ muscle stimulation.

Overall, this study demonstrates that young, growing skeletal muscle, like adult muscle, adapts to consumption of a HFD, but these early adaptive changes in fiber composition and enzyme content do not manifest in a functional improvement in muscle performance or exercise capacity.

## Material and Methods

### Animals

Male C57BL/6J mice (3 weeks old) were obtained from Jackson Labs (Bar Harbor, ME). Mice were given 1 week to acclimatize before the experimental protocol began. Mice were housed in a temperature and humidity controlled facility with a 12/12 h light/dark cycle and were given free access to food and water. All experimental protocols were carried out with approval of the McMaster University Animal Care Committee in accordance with the Canadian Council for Animal Care guidelines.

Mice performed an initial exercise test and were then divided into three groups: sedentary standard chow diet (CON, *n* = 12), sedentary high‐fat diet (HFD, *n* = 12), and exercise standard chow diet (EX, *n* = 11). The standard chow diet contained 25% of energy (kcal/g) from fat, 20% protein, and 55% carbohydrate (LabDiet 5015 Mouse Diet; St. Louis, MO) and the high‐fat diet contained 60% fat, 20% protein, and 20% carbohydrate (D12492; ResearchDiets Inc., New Brunswick, NJ). The diet length (3 weeks) was chosen to coincide with a period of steep growth for the mice (4–7 weeks of age) and to allow for comparison with our previously published adult data (Shortreed et al. 2009; Trajcevski et al. 2013). We also included a group of standard chow diet aerobically trained mice to compare with the expected oxidative changes in the HFD group. Aerobic training has been shown to induce oxidative changes in skeletal muscle resulting in functional benefits to lipid oxidation and exercise performance as we hypothesized would occur in the HFD group. Upon completion of the 3 weeks of exercise training or sedentary behavior, mice from each group were divided into two groups. One group performed a final exercise test and in situ muscle stimulation while the other group was used for the glucose tolerance test and palmitate oxidation measurements (see below).

### Exercise test

Mice were acclimatized to the rodent treadmill (Columbus Instruments, Columbus, OH) prior to exercise testing for 2 days with at least 1 day of rest before the exercise test. On the first day of acclimation, the mice were placed on an unmoving treadmill for 5 min and then speed was increased by 1 m/min every 1 min up to a speed of 10 m/min. On day 2, the mice sat on an unmoving treadmill for 5 min and the speed was then increased by 1 m/min every 30 sec up to a speed of 20 m/min. All acclimation was performed at a 0° incline.

The progressive treadmill exercise test was performed on a treadmill at a 5° incline. The mice ran for 2 min at 10 m/min and the speed was then increased by 1 m/min every 2 min until the animal reached exhaustion. Exhaustion was defined when the mice came off the back of the treadmill twice within 1 min for 10 sec and refused to get back on. The exercise test was performed before the training program began and then again at completion of the 3 week training program.

### Exercise training

Treadmill training was carried out over 3 weeks for 5 days each week. During the first week, mice ran on a 15° incline with a 5 min warm up at 10 m/min after which the speed was increased to 22 m/min for 55 min and an additional 5 min where the speed was increased by 1 m/min each minute and then held at 27 m/min for another 5 min. During the second week, the base speed was increased to 23 m/min with the final 5 min being completed at 28 m/min. For week 3, the base and final speeds were increased by 1 m/min after the initial 5 min warm up at 10 m/min.

### Glucose tolerance test

An intraperitoneal glucose tolerance test (IPGTT) was performed on mice following a 6 h fast. An intraperitoneal injection of glucose was given (1 g/kg body weight) and blood glucose was monitored by tail bleed immediately prior to injection and at multiple time points post glucose injection for 120 min.

### In situ muscle stimulation

Muscle stimulation was performed at least 48 h after the last exercise bout. Mice were anesthetized with an intraperitoneal injection of ketamine/xylazine (100 mg/kg/ 10 mg/kg) and the right gastrocnemius/plantaris/soleus complex (triceps surae) was isolated at the Achilles tendon. The Achilles tendon was then attached to a force transducer and optimal voltage and length were determined. Muscle was stimulated via the sciatic nerve using platinum‐coated electrodes. A fatigue protocol was run on all mice consisting of 5 min of 100 Hz stimulation lasting 100 msec in 1 sec trains. This was followed by 5 min of 300 msec of 100 Hz stimulation in trains of 400 msec. Muscle function testing was performed using a whole mouse test system from Aurora Scientific Instruments (1300A, Aurora Scientific Inc, Aurora, ON, Canada) and data were collected and analyzed with Dynamic Muscle Control and Analysis software (615A, Aurora Scientific Inc). Following muscle stimulation hind limb muscles were removed from mice, frozen in liquid nitrogen, and stored for later analysis.

### Ex vivo palmitate oxidation

Palmitate oxidation was measured in the soleus and extensor digitorum longus (EDL) as described previously (Steinberg and Dyck 2000). Briefly, muscles were dissected tendon to tendon and incubated in a preincubation buffer (modified Krebs–Henseleit buffer with 4% fatty acid‐free BSA, 2 mmol/L pyruvate, and 0.5 mmol/L palmitate) which was warmed to 30°C and gassed with 95% O_2_/5% CO_2_ for 20 min. The preincubation buffer was then replaced with a similar buffer containing [^14^C]palmitic acid for 60 min. Muscles were removed, blotted dry, weighed, frozen in liquid nitrogen, and stored at −80°C for later analysis. ^14^CO_2_ was collected over 90 min following the addition of 1 mL of acetic acid to measure complete palmitate oxidation.

### Western immunoblotting

Gastrocnemius‐plantaris muscle complex was homogenized in RIPA buffer. Protein content was determined using Bradford assay. Muscle lysates were run on 7.5% or 10% SDS‐PAGE gels and transferred to PVDF membranes. Primary antibodies (MitoProfile Total OxPhos Rodent WB Antibody Cocktail, MS604, MitoSciences [1:5000]; FAT/CD36, HPA002018, Sigma (Oakville, ON, Canada) [1:1000]) were incubated overnight at 4°C followed by appropriate HRP secondary (Abcam, Toronto, ON, Canada). Signal was detected using SuperSignal West Femto Maximum Sensitivity Substrate (34095, Thermo Scientific, Waltham, MA). Images were captured and analyzed with Gel Logic 6000 Pro (CareStream Health, Rochester, NY). Equal protein loading was verified using Ponceau staining.

### Muscle histology

Tibialis anterior (TA) and soleus muscles were removed from mice and coated in optimal cutting temperature compound, frozen in liquid nitrogen cooled isopentane, and stored at −80°C until sectioned. Three serial sections of TA and soleus muscles were cut using a cryostat and stained for lipid content, myosin heavy chain (MyHC) expression, and succinate dehydrogenase (SDH) activity. Lipid content was measured using oil red O (ORO). SDH was measured by incubating muscle sections in a buffer consisting of 0.2 mol/L phosphate buffer, 0.2 mol/L sodium succinate, and 0.08 mol/L nitro blue tetrazolium (Sigma, N6876) for 1 h at 37°C. MyHC isoform expression was determined using the protocol of Bloemberg and Quadrilatero (2012). Briefly, muscle sections were blocked in normal goat serum, primary antibody cocktail was added for 2 h (SC‐71, 1:600; BF‐F3, 1:100; BA‐F8, 1:50, Developmental Studies Hybridoma Bank, Iowa City, IO). Sections were then washed and incubated in the appropriate AlexaFluor secondary antibody (Invitrogen) for 1 h, washed, and cover slipped. Alkaline phosphatase (AP) staining (SigmaFAST BCIP/NBT tablets, Sigma‐Aldrich) was performed on another section of TA and soleus to measure capillary content. Images were taken using Nikon Eclipse 90i microscope with a 20× objective (ORO, SDH, AP) or a 10× objective (MyHC) (Nikon, Mississauga, Canada).

The analysis was carried out using Nikon Elements Analysis software tools (Nikon). Individual fibers from TA and soleus sections were manually circled for ORO and SDH to measure density of staining and fiber cross‐sectional area. Circled fibers were matched between serial sections that had been stained for MyHC to determine fiber type. Capillary density was determined by thresholding and defined as the area of stained AP‐positive capillaries relative to total muscle area.

### Statistics

Body mass, IPGTT, exercise test, and in situ fatigue test were assessed using repeated measures two‐way ANOVA with Bonferroni post hoc test. All other measures were assessed using a one‐way ANOVA with Bonferroni post hoc test. Significance was set at a *P* ≤ 0.05. All statistical analysis was performed using GraphPad Prism 5 software (La Jolla, CA). Data are presented as means ± standard error of the mean (SEM).

## Results

### Body composition and glucose tolerance

**Figure 1.**
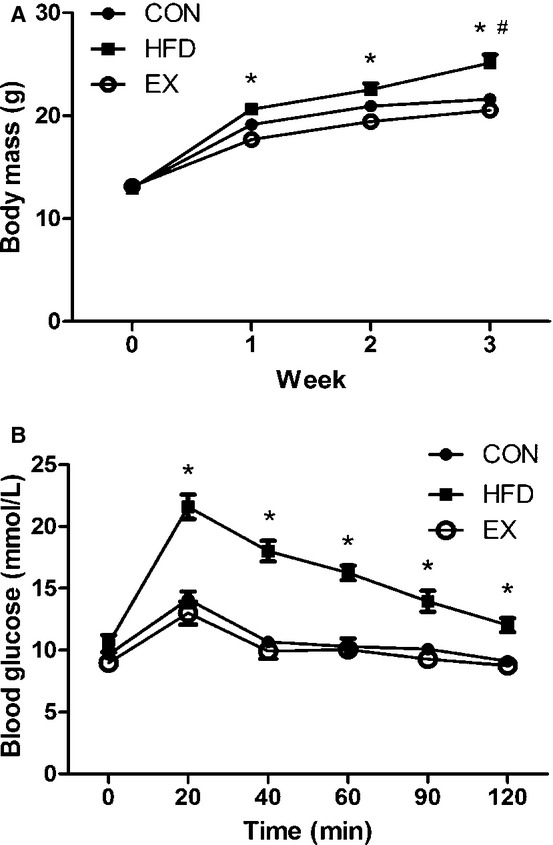
Body mass and glucose tolerance test. (A) Body mass of mice over 3 weeks (*HFD vs. EX, #HFD vs. CON,* P* < 0.05). (B) Intraperitoneal glucose tolerance test (*HFD vs. CON and EX,* P* < 0.05). Data are mean ± SEM,* N* = 5–6.

Three weeks of HFD consumption resulted in a significant increase in body mass compared to both CON and EX mice. Exercise training had no effect on body mass compared to CON mice, but after just 1 week of training, body mass of EX was lower than HFD mice (Fig. 1A). The increase in body mass of the HFD mice was likely due to the significant increase in fat mass, as muscle mass was not different from the CON group (Table 1). While exercise training did not affect fat mass, it did significantly lower muscle mass compared to the sedentary groups (CON and HFD).

**Table 1.** Epididymal fat mass and muscle masses among the various groups

Group mass (mg)	CON	HFD	EX
Epididymal fat	160.8 ± 9.3	460.3 ± 43.4^1^	138.8 ± 6.9
Gast‐Plantaris	129.8 ± 2.1	129.0 ± 3.8	120.3 ± 1.9^2^
Soleus	8.4 ± 0.4	9.7 ± 0.7	7.0 ± 0.2^3^
Tibialis anterior	36.7 ± 1.0	38.7 ± 1.2	34.9 ± 0.3^3^

Data are mean ± SEM, *N* = 6–12. *P* < 0.05. Gast‐Plantaris refers to the gastrocnemius‐plantaris muscle group.

^1^ Denotes different from CON and EX.

^2^ Different from CON.

^3^ Different from HFD.

Fasted basal glucose levels were not different between groups. In response to a glucose challenge, HFD mice displayed significantly higher blood glucose levels (vs. CON and EX) within 20 min and this significant elevation in blood glucose levels persisted throughout the test (Fig. 1B).

### Skeletal muscle morphology

**Figure 2.**
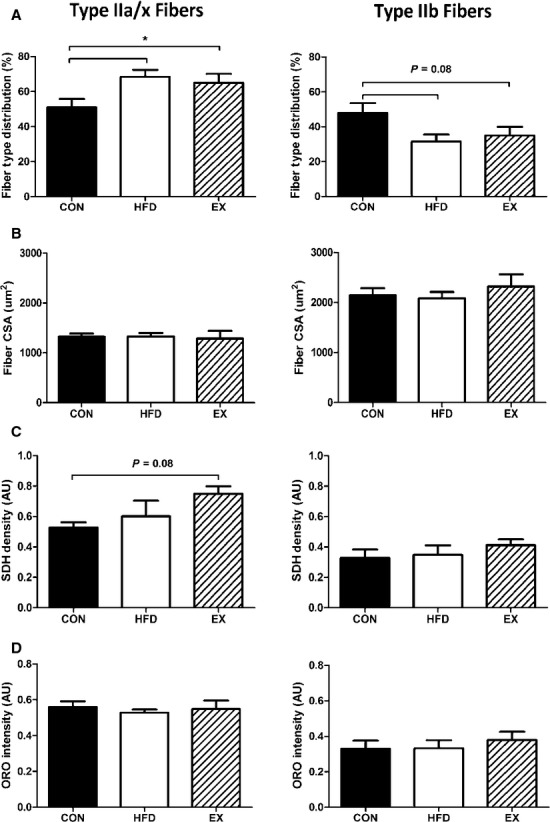
Tibialis anterior morphology. (A) Fiber‐type distribution based on myosin heavy chain isoform staining. *HFD versus CON. (B) Fiber cross‐sectional area. (C) Succinate dehydrogenase density. (D) IMCL content was not affected by any treatment. Data are mean ± SEM,* N* = 5–6.

To determine the adaptive changes occurring in skeletal muscles with HFD consumption or exercise training, we assessed fiber‐type distribution, fiber cross‐sectional area (CSA), SDH density, and intramyocellular lipid (ORO) content. It is important to note that the oxidative capacity of mouse skeletal muscle fibers is type IIa>IIx/I>IIb. Type I fibers were not included in the TA muscle analysis due to their minimal presence in that muscle (Burkholder et al. 1994). As we were interested in changes in oxidative versus glycolytic fibers in the TA muscle, we grouped oxidative IIa and IIx fibers together for this analysis, similar to previous work (Shortreed et al. 2009). The TA muscle demonstrated a significant effect of HFD and EX highlighting the shift to a more oxidative fiber‐type distribution (Fig. 2A). There was a nonsignificant trend (*P* = 0.08) for a decrease in type IIb fibers in HFD and EX groups compared to CON (Fig. 2A). Fiber CSA was not affected by diet or exercise in any fiber type (Fig. 2B). SDH density, as a surrogate measure of mitochondrial content, showed a nonsignificant trend (*P* = 0.08) of an increase with EX in oxidative IIa/x fibers. The glycolytic type IIb fibers had no changes in SDH density (Fig. 2C). Importantly, when one considers the shift in fiber‐type distribution toward more IIa/x and a concomitant shift in SDH density in these fiber types, the overall outcome would be significantly more mitochondria per muscle in both HFD and EX muscles. IMCL content, as assessed by ORO, was not affected by any treatment in any fiber type (Fig. 2D).

**Figure 3.**
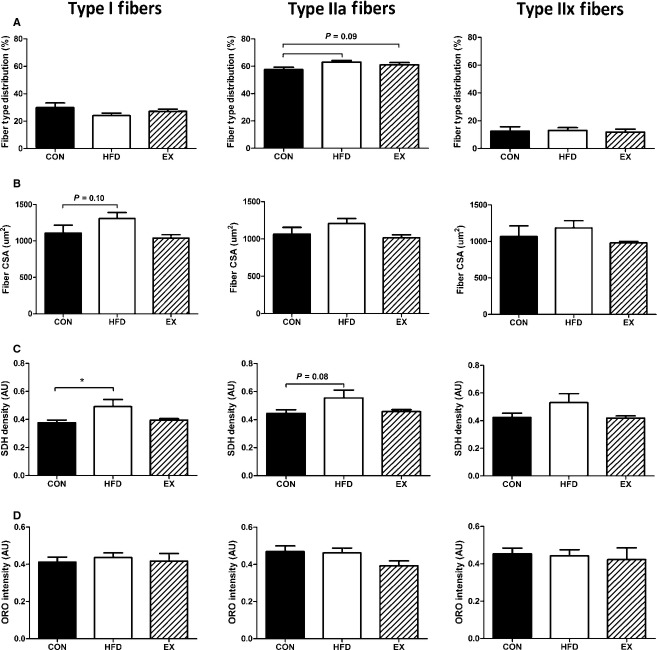
Soleus muscle morphology. (A) Fiber‐type distribution based on myosin heavy chain isoform staining. (B) Fiber cross‐sectional area. (C) Succinate dehydrogenase density was increased with exercise in type I fibers (*HFD vs. CON,* P* < 0.05). (D) IMCL content was not affected by any treatment. Data are mean ± SEM,* N* = 5–6.

The soleus muscle is a highly oxidative muscle and has virtually no type IIb fibers (Burkholder et al. 1994). Thus, this fiber type was excluded from the analysis. There was a trend (*P* = 0.09) for a shift to greater percentage of IIa fibers in the HFD and EX groups compared to CON mice, whereas type I and IIx fiber‐type proportions were unaffected by any treatment (Fig. 3A). Fiber CSA were unaffected by treatment regardless of fiber type. However, a trend (*P* = 0.10) for an increase in fiber CSA with HFD in type I fibers was observed (Fig. 3B). The density of SDH was increased in type I fibers with HFD compared with CON and there was a nonsignificant (*P* = 0.08) increase in SDH with HFD in type IIa fibers. Type IIx fibers showed no changes (*P* = 0.14) in SDH content with any treatment (Fig. 3C). IMCL content was not affected by any treatment in any fiber type (Fig. 3D).

### Functional measures

**Figure 4.**
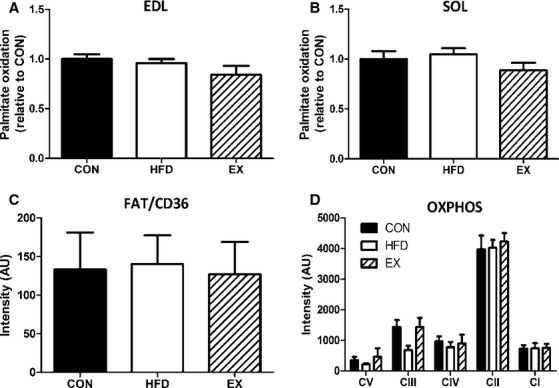
Palmitate oxidation relative to CON group was not affected by HFD or EX. (A) EDL palmitate oxidation. (B) Soleus palmitate oxidation. Data are mean ± SEM,* N* = 5–6. (C) Western blotting demonstrated no effect of group (*N* = 3/group) with respect to FAT/CD36 levels. (D) Ox Phos Western blotting also demonstrated no effect of HFD or EX compared to CON (*N* = 5–6/group).

Whole muscle ex vivo palmitate oxidation was assessed to determine if lipid oxidation rates were altered with HFD or exercise training. Despite the aforementioned morphological results indicating an oxidative shift in the HFD and EX groups, palmitate oxidation rates were not different in the glycolytic EDL (Fig. 4A) or oxidative soleus (Fig. 4B) muscles between any groups. These functional measures were consistent with the lack of change in FAT/CD36 and oxidative phosphorylation (Ox Phos) proteins as detected by Western blotting (Fig. 4C and D).

**Figure 5.**
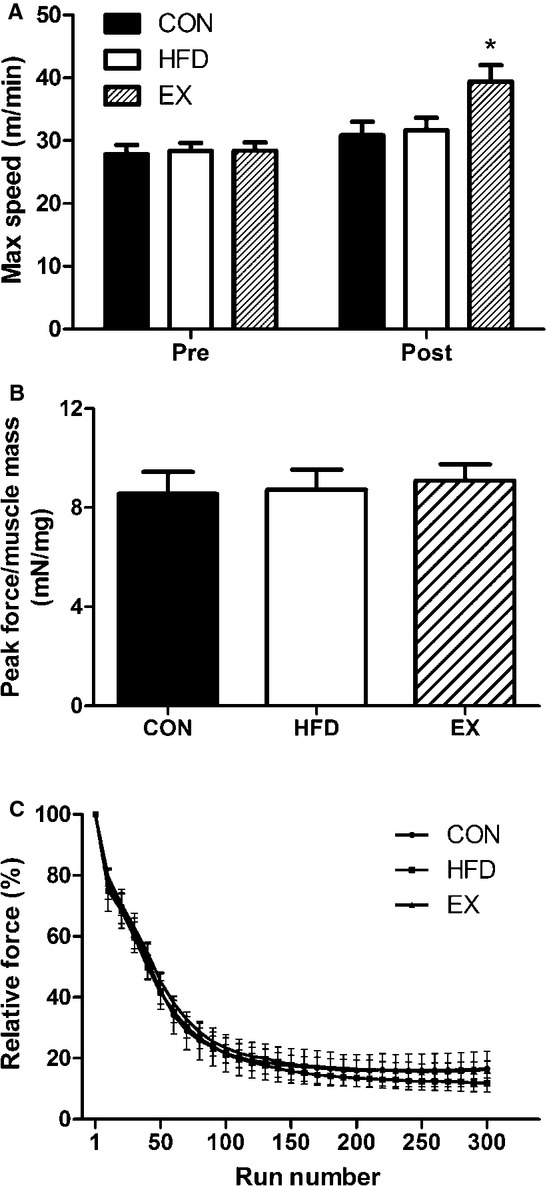
Exercise test performance and in situ force measurement. (A) Maximal speed reached on treadmill exercise test before and after 3 weeks training or diet intervention (*EX vs. CON and HFD post intervention, *P* < 0.05). (B) Peak tetanic force of triceps surae normalized to muscle mass. (C) Relative force normalized to peak initial force during first 5 min of fatigue protocol. Data are mean ± SEM,* N* = 5–6.

A progressive treadmill exercise test was performed before and after 3 weeks of exercise training or diet intervention to assess whole body exercise capacity. The maximal speed attained during the test was not different between any of the groups at baseline. After 3 weeks of treatment (diet or exercise), maximal speed was higher in EX versus CON and HFD groups (Fig. 5A) with no difference between the CON and HFD. Maximal time of the exercise test (time to fatigue) followed a similar pattern to maximal speed, with no difference between groups at baseline, greater time to fatigue in the EX group (relative to HFD and CON), and no difference between CON and HFD post treatment.

To determine if the in vivo exercise test results were specifically due to changes in skeletal muscle functionality, we performed an in situ muscle fatigue test on the triceps surae complex. There was no difference in absolute peak force or normalized peak force between any of the groups (Fig. 5B). The rate of fatigue was also not affected by exercise training or HFD consumption (Fig. 5C, only first 5 min shown). The run number to reach 50% of initial force (CON: 44 ± 8, HFD: 38 ± 5, EX: 45 ± 2) or 25% of initial force (CON: 107 ± 28, HFD: 89 ± 11, EX: 119 ± 33) was also not different between groups.

### Capillary density

**Figure 6.**
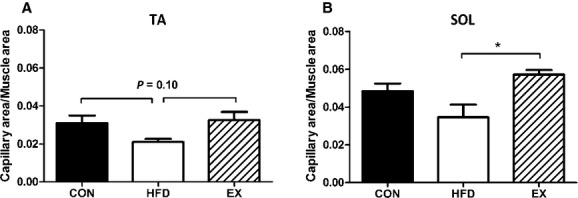
Capillary area relative to muscle area. (A) Tibialis anterior capillary content was not different with any treatment. (B) Soleus capillary content was lower in HFD compared to EX (*HFD vs. EX,* P* < 0.05). Data are mean ± SEM,* N* = 5–6.

We investigated capillary content as a possible explanation for the difference in performance on the exercise test between the groups. There was no significant difference in capillary content in the TA muscle between any groups, although there was a nonsignificant trend (*P* = 0.10) for it to be lower in the HFD group compared to CON and EX (Fig. 6A). Capillary content of the soleus muscle was significantly lower in the HFD mice compared to the EX group (Fig. 6B).

## Discussion

In this study, we investigated whether the oxidative shift in skeletal muscle morphology that occurs in adult mice with short‐term consumption of a HFD (Turner et al. 2007; Hancock et al. 2008; de Wilde et al. 2008; Trajcevski et al. 2013) occurs similarly in muscle of young, growing mice. We then investigated if these morphological changes in skeletal muscle composition manifested as a functional improvement, as determined by a treadmill exercise test and in situ muscle contractile function testing. Despite higher metabolic rates associated with the growth phase (FAO/WHO/UNU 2004), our results show that only 3 weeks of HFD is enough to cause significant increases in body mass (due to large gains in fat mass) and whole‐body insulin resistance in young, growing mice. At the level of skeletal muscle, the TA muscles of HFD mice showed a shift to a more oxidative fiber type (less glycolytic type IIb and more oxidative type IIa/x) and a trend for more mitochondria (as assessed by SDH) compared to the standard chow controls. In the soleus muscles, a primarily oxidative muscle, more SDH and an increased proportion (*P* = 0.09) of the oxidative IIa fibers were observed in response to HFD compared to CON.

The relatively short consumption of a high‐fat diet resulted in significant changes to body composition as seen by the 2.9‐fold increase in epididymal fat mass compared to normal diet young mice. This increase in fat mass is slightly less (2.9‐fold vs. 3.5‐fold) than what we have seen when adult mice were placed on the same diet for the same duration (Trajcevski et al. 2013). Young mice may be accumulating fat mass at a slightly lower rate than adult mice over 3 weeks of high‐fat diet consumption due to a higher rate of resting energy expenditure as has been seen in human adolescents relative to adults (FAO/WHO/UNU 2004). When the HFD is continued for an additional 5 weeks, young mice gain epididymal fat mass at a higher rate than adult mice such that young mice have a ~fivefold increase in epididymal fat mass relative to their age‐matched CON counterparts (K. E. Trajcevski, unpubl. obs.), whereas adults remain at an approximately threefold increase in fat mass compared to CON (Shortreed et al. 2009). C57BL/6J mice show steep gains in body mass between 3 and approximately 6 weeks of age after which the rate slows (The Jackson Laboratory 2007). The current study was carried out during the steep growth phase suggesting that the elevated basal metabolic rate associated with growth was a primary mechanism in the attenuation of fat mass accumulation. Consistent with this, the absolute levels of palmitate oxidation in the CON animals of this study were higher than those recorded using the same techniques in our previous studies using adult mice (Trajcevski et al. 2013).

Whole muscle palmitate oxidation rates were not affected by treatment (HFD or EX) despite the morphological (oxidative) shifts that occurred. This lack of change in palmitate oxidation rate differs from what we have observed in adult muscle where EDL palmitate oxidation rate was increased with 3 weeks of HFD (Trajcevski et al. 2013). However, adult normal diet mice had lower palmitate oxidation rates than our young mice (as noted above) and the consumption of a HFD in the adult mice elevated palmitate oxidation rates to levels consistent with those measured in the young mice of the current study. In addition to the present study, Bonnard et al. (2008) report that 4 weeks of a high‐fat high‐sugar diet did not alter gastrocnemius muscle oxidation rates in mice who began the diet at 5 weeks of age. We would speculate that the high basal level of palmitate oxidation in the young mice was primarily responsible for the lack of IMCL accumulation in the muscle; a finding supported by the observation that 3 weeks of HFD did not result in increased IMCLs in adult mice (whose lipid oxidation rates were similar to that of young mice) (Trajcevski et al. 2013). As well, the oxidative shift reported here may have prevented a build up IMCLs as oxidative fiber types are more readily able to utilize lipids as a fuel. Oxidative fibers also store more IMCLs than glycolytic fibers, thus the fiber‐type shift in the TA would result in greater lipid storage capacity and the increased fiber size in the soleus fibers would result in an overall greater storage capacity for the muscle (without affecting the per fiber values reported here). When high‐fat diets are sustained for longer durations (6–16 weeks) in adult mice they do cause significantly lower oxidation rates and increased lipid accumulation (Chanseaume et al. 2007; Bonnard et al. 2008; Shortreed et al. 2009). We hypothesize that the same decrement in lipid oxidation rate occurs in young mice fed a long‐term HFD given that these young HFD mice do display significant increases in ORO density when the diet persists for a total of 8 weeks (data not shown). A limitation of the palmitate oxidation results presented here are that they were undertaken in the basal state. Thus, just because the capacity to oxidize fat in a resting muscle may have been increased would not necessarily translate into increased palmitate oxidation. Providing a stimulus to elicit maximal palmitate oxidation (e.g., uncouple mitochondrial respiration) may uncover differences between groups not detected at the basal level in the present work. Although this measure was not undertaken in this study, we would speculate that if there were major changes in this measure that the stress induced by exercise would have been sufficient to elicit this response (and therefore manifest as a functional difference) though future studies would be needed to assess this. It is also interesting to note that no change in fat transporters (FAT/CD36) were detected in the muscle of the HFD mice suggesting that while more fat was available, the ability to transport it into the muscle may have been limiting.

We had hypothesized that the oxidative shifts in skeletal muscle would result in functional benefits on an exercise test and fatigue resistance during an in situ test, however, this did not occur. Miller et al. (1984) have shown that adult rats were able to run on a treadmill for a longer duration after consuming 1 or 5 weeks of a low‐carbohydrate, high‐fat diet. Lee et al. (2001) also showed similar improvements to Miller et al. (1984) with 8 weeks of HFD in sedentary rats. The exercise tests used in the previous studies (Miller et al. 1984; Lee et al. 2001) were performed at constant speeds and our test used incremental increases in speed which may have increased the reliance on carbohydrate fuel sources as the speed reached high levels in contrast to the constant speed test which would have relied more on lipids as a fuel source.

Another possible explanation for the lack of change in exercise capacity could be the decreased capillary density observed in the HFD mice. In contrast to our findings in young mouse muscle, previous studies have shown no change in capillary density with longer (8 and 12 week) duration HFD (Li et al. 2007; Roudier et al. 2009), and one study reported an increase in capillary density with 19 weeks of a HFD (Silvennoinen et al. 2013). Differences in diet length and fat content (45% vs. 60%) may account for the inconsistent results between our study and those in the literature. In support of this, we do not detect significant differences in capillary density between CON and HFD young mice when they consume the diet for 8 weeks (data not shown). The combination of a higher capillary density (vs. HFD) and oxidative shift in skeletal muscles of the EX mice may account for their improved exercise capacity. While the HFD mice had a shift in oxidative capacity their lower capillary density may not have allowed them to adequately supply the oxidative fibers with the oxygen needed to take advantage of this shift and thus resulted in no improvement to exercise capacity. However, the oxidative shift likely compensated for the decreased capillary density and resulted in the HFD mice maintaining a similar exercise capacity to the CON mice.

Taken together, the present work demonstrates that young, growing muscles exhibit early oxidative shifts in response to high‐fat diet consumption. However, these changes do not translate to functional benefits in lipid oxidation, muscle fatigue resistance, or whole body exercise capacity. In addition this work serves to highlight the importance to performing functional tests as changes to morphology do not always translate into changes in physiology.

## Acknowledgments

The authors have no conflict of interest to report. The myosin heavy chain antibodies (BA‐F8, SC‐71, BF‐F3) developed by Stefano Schiaffino were obtained from the Developmental Studies Hybridoma Bank developed under the auspices of the NCHD and maintained by The University of Iowa, Department of Biology, Iowa City, IA 52242.

## Conflict of Interest

None declared.
